# Improving bioenergy recovery from municipal wastewater with a novel cloth-filter anaerobic membrane bioreactor

**DOI:** 10.3389/fbioe.2023.1242927

**Published:** 2023-11-22

**Authors:** Neriamara Martins-West, Ana Martin-Ryals, Bryan Maxwell, Lance Schideman

**Affiliations:** ^1^ Illinois Sustainable Technology Center, University of Illinois at Urbana-Champaign, Champaign, Illinois, United States; ^2^ University of Florida, Agricultural and Biological Engineering, Gainesville, FL, United States

**Keywords:** anaerobic digestion, AnMBR, municipal wastewater, decarbonization, cloth filter, bioenergy, membrane

## Abstract

Anaerobic membrane bioreactors (AnMBR) have been used for treating high-strength industrial wastewater at full-scale and the potential to use them for mainstream municipal wastewater treatment presents an important opportunity to turn energy-intensive plants into net-energy producers. However, several limitations of the AnMBR technology have prevented their adoption in the municipal wastewater industry, namely, high membrane cleaning energy demand and low membrane flux. This study demonstrated a novel AnMBR configuration that uses a commercially available cloth filter technology to address the key limitations of cleaning energy and membrane flux. The cloth filter anaerobic membrane bioreactor (CFAnMBR) is comprised of an anaerobic fixed-film bioreactor coupled with a cloth filter membrane with nominal pore size of 5 µm. The pilot CFAnMBR was operated for 150 days through the winter at a municipal wastewater plant in central Illinois (minimum/average influent temperature 5/13°C). The CFAnMBR increased membrane flux by more than 2 orders of magnitude (3,649 ± 1,246 L per meter squared per hour) and reduced cleaning energy demand by 78%—92% (0.0085 kWh/m^3^) relative to previously reported AnMBR configurations. With the CFAnMBR, average chemical oxygen demand and total suspended solids removal were 66% and 91%, respectively, and were shown to be increased up to 88% and 96% by in-line coagulant dosing with ferric chloride. Average headspace methane yield was 154 mL CH_4_/g COD_removed_ by the end of the study period with influent temperatures of 11°C± 4°C. The CFAnMBR resolves major limitations of AnMBR technology by employing a commercially-available technology already used for other municipal wastewater treatment applications.

## 1 Introduction

Current wastewater treatment relies primarily on conventional activated sludge (CAS) for aerobic degradation of organics into carbon dioxide (CO_2_). Although this process is robust and provides a good-quality effluent, CAS is an energy-intensive treatment process that produces large amounts of sludge and has a considerable carbon footprint. Moreover, it is a double negative for energy efficiency because it dissipates the organic energy content of wastewater and requires substantial energy input for aeration, which accounts for 40%–60% of the total energy demand for a typical wastewater treatment plant (WWTP) (Vinardell et al., 2020). It is estimated that wastewater treatment plants consume 30 billion kWh/year, or about 1%–3% of the US electricity demand (Pabi et al., 2013), and thus contribute significantly to the cost of treatment and the greenhouse gas emissions from electricity production.

Shifting from aerobic to anaerobic treatment of municipal wastewater presents a powerful opportunity to turn energy-intensive municipal wastewater treatment plants into resource recovery operations and net-energy producers (Li and Yu, 2016). The economic value of energy and nutrients in wastewater can help offset the cost of wastewater treatment and avoid adverse environmental impacts (Song et al., 2018). In fact, typical domestic wastewaters have the potential of producing 1.93 kWh/m^3^ from organic oxidation and could offset 0.79 kWh/m^3^ required to produce fertilizers (McCarty et al., 2011). Through anaerobic digestion, nutrients are converted to chemically available forms (e.g., ammonia and phosphate) that can be recovered via physicochemical processes such as coagulation, flocculation, filtration, and ion exchange.

In recent years, the interest in using membrane technologies in conjunction with anaerobic reactors has increased considerably. Anaerobic membrane bioreactors (AnMBRs) combine anaerobic biological treatment with filtration so that biomass can be fully retained within the reactor, which thoroughly decouples the hydraulic retention time (HRT) and the solids retention time (SRT). This separation is incredibly impactful when treating municipal wastewater, typically characterized by high volumetric flow rate, low organic strength, and a significant amount of particulate organics that need to be hydrolyzed before being converted into the final products of anaerobic digestion (Martinez-Sosa et al., 2011). The hydrolysis process becomes a rate-limiting step of anaerobic treatment, especially at low temperatures, requiring either heating of reactors or increased SRT. Both of these options become economically unfeasible for municipal wastewater treatment because heating the entire wastewater flow would incur a very large cost and increasing SRT would require additional capital expenditure for large reactors and land utilization. Conversely, AnMBRs can provide long SRTs with small reactor sizes due to the decoupling of HRT and SRT provided by the membrane, which allows water to pass through quickly while retaining particulate/colloidal organics in the reactor until they can be degraded (Martinez-Sosa et al., 2011). This decoupling facilitates the use of very long SRTs without increasing reactor volume, which facilitates the survival of slow-growing microbes like Methanogenic Archaea (MA) and sulfate-reducing bacteria (SRB) that are also retained by the membrane to achieve high biogas production and sulfate reduction rates. AnMBRs also provide low effluent concentrations of suspended solids, and rejection of bacteria and viruses in the treated effluent (Gao et al., 2012).

Despite the advantages of AnMBRs, application of this technology for municipal wastewater treatment has been limited to pilot-scale due to several practical challenges (Robles et al., 2018). In particular, the high operating costs and energy inputs for membrane fouling control is the most noted drawback. AnMBRs also have relatively low permeate flux rates, high costs for the membrane, and high effluent concentrations of nutrients and dissolved methane (Kim et al., 2011; Martinez-Sosa et al., 2011; Gouveia et al., 2015a; Pretel et al., 2016; Shin and Bae, 2018; Vinardell et al., 2020). Additionally, post-treatment processes to recover nutrients and dissolved methane are not well documented. Since membrane fouling is the leading cause of membrane flux decline over time (Vinardell et al., 2020), employing appropriate fouling control strategies becomes critical to improve membrane flux, which directly impacts both capital and operating expenditures. The process may be physical, chemical, or biological. The most common physical control used in AnMBRs is the application of shear stress to limit foulant deposition, which is generally achieved by high cross-flow velocities in side-stream AnMBRs, while biogas and particle sparging, and rotating membrane are commonly used for submerged AnMBRs (Shin and Bae, 2018).

The addition or formation of granular materials has been considered to mitigate membrane fouling in AnMBRs. The addition of granular or powdered activated carbon has been shown to reduce membrane fouling effectively by several studies (Aslam et al., 2014, 2017; Martinez-Sosa et al., 2012; Shin et al., 2014). The particles provided mechanical scouring of the membrane surface, and the adsorbents served as support media for biofilm growth and reduced the viscosity of the activated sludge. The addition of ferric chloride as a coagulant to increase particle sizes was shown to retard membrane fouling by over 90 days (Dong et al., 2015). Fluidized systems have been reported to be effective in controlling membrane fouling and have lower energy consumption compared to cross-flow systems (Seib et al., 2016a; Seib et al., 2016b). Shear forces developed through mechanical movement of the membrane have also been tested to reduce fouling. Several membrane configurations, such as tubular, hollow fiber, and flat-sheet discs, have been tested in submerged rotating AnMBRs with varying degrees of success in terms of improving filtration performance or reducing transmembrane pressue (TMP) (Maaz et al., 2019). However, the above-mentioned fouling mitigation techniques still require significant energy, which often dominates the total operating costs and has substantial environmental impacts. While membrane costs have decreased and fouling mitigation techniques have improved, the low flux rates of most AnMBRs still result in higher capital and operating costs than CAS without tertiary treatment (Ng et al., 2020).

The objective of this study was to develop and demonstrate a novel integrated cloth-filter AnMBR (CFAnMBR) system with the potential to treat municipal wastewater at lower cost and energy demand compared to current AnMBR technology while also providing effluent quality and nutrient removal comparable to CAS. The hypothesis behind the proposed novel CFAnMBR was that a membrane with a larger pore size could operate at much higher flux rates than conventional AnMBRs and still capture and concentrate the slow-growing anaerobic microorganisms but at a much lower energy input and cost. The CFAnMBR design included the use of plastic support media in the anaerobic treatment tank upstream of the cloth-filter to provide surface area for biofilm growth and reduce the solids load impinging on the cloth-filter, thus reducing the energy needed for filtration and fouling mitigation.

## 2 Materials and methods

### 2.1 CFAnMBR design and operation

Testing of the cloth filter anaerobic membrane bioreactor (CFAnMBR) was performed at the Urbana Champaign Sanitary District in Urbana, Illinois, USA over 150 days between October 2020 and March 2021. The CFAnMBR was located on site of a wastewater resource recovery facility (WRRF) to perform testing on real municipal wastewater influent after screening and grit removal. The average characteristics of the influent wastewater over the study period study are summarized in Table 1.

**TABLE 1:** Average characteristics of CFAnMBR influent over the study period.

Parameter	Average ± Std. Dev
Influent Flow Rate (m^3^/h)	0.25 ± 0.1
TSS (mg/L)	400 ± 205
COD (mg/L)	487 ± 95
SO_4_-S (mg/L)	27 ± 4
Total ammoniacal nitrogen (mg/L)	19 ± 7
Total PO_4_-P (mg/L)	4.5 ± 1.7
pH	6.9 ± 0.5
Temperature (°C)	14.4 ± 4.0

**FIGURE 1:**
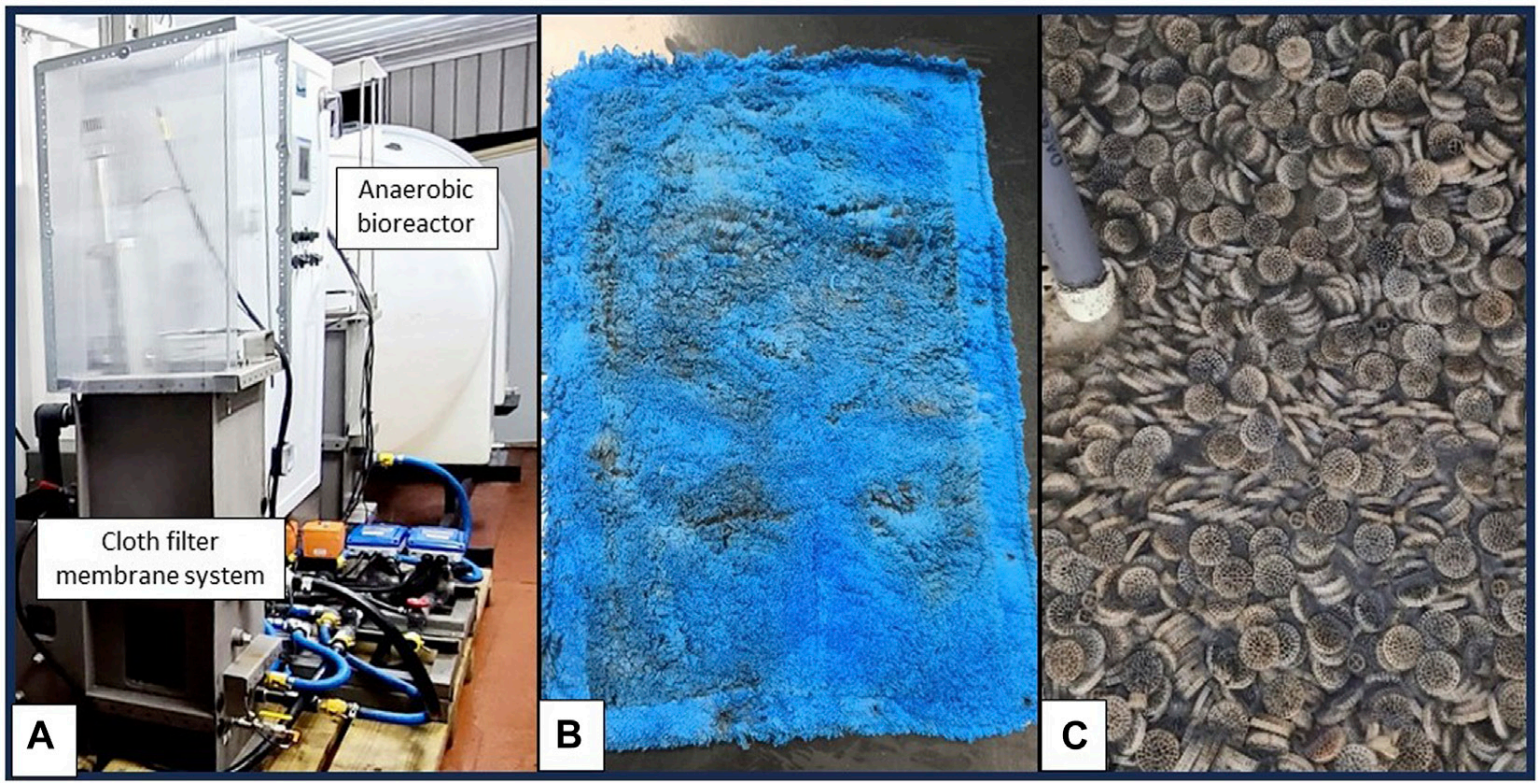
Photos of the pilot-scale cloth filter anaerobic membrane bioreactor with: **(A)** polyethylene tank filled serving as the anaerobic bioreactor, with skid-mounted cloth filter membrane receiving effluent from the bioreactor; **(B)** cloth filter material (nominal 5 µm pore size) serving as the membrane for solids retention; **(C)** colonized plastic biofilm support media following microbial acclimation period using synthetic wastewater.

The small pilot-scale CFAnMBR system was composed of two main components which included 1) an anaerobic fixed-film bioreactor (AFFB) and 2) a cloth filter membrane (Figure 1). The AFFB used a polyethylene tank with total volume of 3.5 m^3^ and a working volume of 3.2 m^3^, with the remaining volume of the sealed tank used as headspace for biogas accumulation. The average daily flow rate over the study period was 0.25 m3/h, which corresponds to a nominal hydraulic retention time (HRT) of ∼12.8 h. The target organic loading rate (OLR) was 1 kg COD/m^3^/day. The HRT was occasionally adjusted based on the influent COD concentration to provide a more uniform organic loading rate. Water entered the AFFB through a perforated PVC manifold (1″ PVC) at the tank inlet and exited through a perforated PVC manifold (1” PVC) at the tank outlet. Water exited the AFFB via gravity-flow and was then directed to the cloth filter membrane. The pilot CFAnMBR was located within an insulated shipping container. Freezing of the bioreactor during low winter temperatures was prevented using space heaters.

A webbed disk plastic media with 2.5 cm diameter (Veolia Water Technology, Boston, MA, US) was added to the AFFB as a biofilm support media to increase anaerobic biomass retention and reduce solids loading on the downstream cloth filter membrane. The tank was filled with 1.2 m^3^ of pre-colonized, plastic biofilm support media. The AFFB and plastic media were inoculated with a mix of suspended and fixed film biomass using mesophilic anaerobic sludge collected from the Urbana Champaign Sanitary District and acclimated to ambient temperature. Pre-colonization of the plastic media prior to use in the AFFB was achieved by feeding the media with synthetic wastewater made with ground dog food for high COD. Prior to the AFFB, two 200 L drums were filled with the biofilm support media and batch fed at increasing organic loading rates. When the support media showed a high degree of colonization (based on biofilm accumulation, COD removal and biogas production), the plastic media and suspended biomass were transferred to the AFFB to receive a continuous inflow of the municipal wastewater (Table 1).

The cloth filter membrane consisted of a skid-mounted unit with automated controls and monitoring system provided by Aqua-Aerobic Systems, Inc (Loves Park, IL, US), a commercial provider of cloth filter systems typically used for tertiary filtration of municipal wastewater. The cloth filter skid contained a vertically mounted cloth membrane (nominal 5 µm pore size, 0.047 m^2^ of surface area) for solids filtration. Due to the larger pore size of the membrane, water was able to flow through the membrane via gravity flow. To manage fouling of the membrane, the membrane is periodically cleaned using an automated procedure whereby a backwash shoe passes over the cloth membrane while a vacuum is applied using a centrifugal pump (Baldor Reliance Super E Motor, 1.5 horsepower). The solid-liquid mixture collected during backwash is returned to the AFFB, thereby increasing the SRT of the CFAnMBR system. A regular solids waste cycle of the cloth filter basin (every 1—2 h) also returns settled solids to the AFFB.

### 2.2 Cleaning energy demand

The major energy inputs in the CFAnMBR system for fouling control were the energy for backwash and solids waste for returning solids in the AFFB effluent back to the AFFB. The main operating conditions related to the filtration process considered to evaluate the energy consumption of the CFAnMBR were as follows: vacuum pressure during backwash: 3.9 kPa–10.6 kPa; backwash flow rate: 1.8 m^3^/h—2.7 m^3^/h; TSS entering the cloth-filter tank: 41 mg/L—6,188 mg/L; and solids recycling flow rate: 1.4 m^3^/h—2.3 m^3^/h. To estimate the energy inputs, pump shaft power requirement was determined based on the flow rate and head of the pump for the solids waste system, and flow rate and vacuum pressure in the backwash system using the power equation for pumping (Eq. 1):

$$Pw=\frac{Q\times H\times g\times \rho }{\eta }=\frac{Q\times P}{\eta }$$ (1)

where P_w_ = power (watts), Q = flow rate (m^3^/s), H = hydraulic head m), g = gravitational acceleration constant (m/s^2^), *ρ* = density of water (1,000 kg/m^3^), *η* = pump efficiency (65%), and P = vacuum pressure (Pa). The energy demand of the solids waste (E_SW_) and backwash (E_BW_) could then be calculated based on the pump shaft power and operating time, T, and normalized by the volume of treated water (V_permeate_) as shown in Eq. 2 and (Eq. 3):

$$E_{SW}=\frac{\left({Pw}_{SW}\times T_{SW}\right)}{V_{permeate}}$$ (2)

$$E_{BW}=\frac{\left({Pw}_{BW}\times T_{BW}\right)}{V_{permeate}}$$ (3)

The main component of the solids waste/recirculation system was the waste pump. Energy consumption by the backwash system consisted of a backwash pump and a drive motor for operating the backwash shoe (E_Drive_) sized at ¼ of the size of the pump (e.g., for a 20 HP backwash pump, a 5 HP drive motor is required at full scale, Eq. 4).

$$E_{Drive}=0.25E_{BW}$$ (4)

The total cleaning energy demand (E_D_) in kWh/m^3^ was estimated by Eq. 5.

$$E_{D}=E_{\text{SW}}+E_{\text{BW}}+E_{\text{Drive}}$$ (5)

Hydraulic flux of the membrane (L/m^2^/h, LMH) was calculated based on the CFAnMBR daily flow volume minus the total daily liquid volume returned to the AFFB during backwash and solids waste.

### 2.3 Analytical methods

Liquid grab samples were collected for AFFB effluent (i.e., mixed liquor) prior to cloth filter and cloth filter permeate. Flow composite samples were collected for the influent to account for variability in wastewater chemistry over 24 h. Before collecting influent and AFFB effluent samples, at least 500 mL were discarded to avoid sampling of stagnant water or accumulated solids in the pipes. The following water chemistry parameters were analyzed according to Standard Methods for the Examination of Water (APHA 2005): total solids (TS), total suspended solids (TSS), sulfate (SO_4_^2-^–S), ammonia, total phosphorus (TP), and chemical oxygen demand (COD). Soluble and total COD were differentiated by filtration, where soluble COD was filtered using 0.45 micron syringe filters prior to analysis. Commercial test kits from Hach and Hanna Instruments were used for chemical tests. Samples were analyzed in duplicate and averaged for each analysis. Water temperature was obtained from a submerged thermometer installed in the cloth-filter tank, and pH was measured using a benchtop meter.

A wet tip gas meter (Wet Tip Gas m, Nashville, TN) was used to measure biogas production. The wet tip gas meter was calibrated periodically to quantify the gas volume required for a tipping event. Biogas samples from the headspace of the bioreactor were collected biweekly in 1 L gas sampling bags (Restek Corp., Centre County, PA), and methane content of the headspace biogas was determined using gas chromatography. The methane concentration of dissolved gases in bioreactor effluent were also determined using a head-space method (Yeo et al., 2015; Yeo and Lee, 2013). Briefly describing, 5 mL permeate was collected using a syringe. The permeate was immediately injected into a 10 mL vacutainer (BD Corp., Franklin Lakes, NJ) and then purged with N_2_. The vial was then shaken with an orbital shaker at 100 rpm for 2 h at room temperature (21°C), allowing thermodynamic equilibrium of methane molecules between the liquid and gas phases. The gas in the headspace of the vial was then collected with a gas-tight syringe (Hamilton Company, Reno, NV). Dissolved and headspace biogas methane concentrations were analyzed using a gas chromatograph HP, Model 5,890 series II (Hewlett Packard Enterprises, Palo Alto, CA) with a thermal conductivity detector (TCD). The carrier gas was helium at a flow rate of 30 mL/min. The injector, oven, and detector temperatures were 200°C, 35°C, and 200°C, respectively. Dissolved methane concentration was calculated using Eq. 6:

$${CH}_{4\left(aq\right)}=\left({CH}_{4}\times P\times K_{CH4}\times {MW}_{CH4}\right)+\frac{\left({CH}_{4}\times V_{hs}\times {MW}_{CH4}\times T_{0}\right)}{V_{W}\times \left(22.4\frac{L}{mol}\right)\times T_{a}}$$ (6)

Where ${CH}_{4\left(aq\right)}$ = concentration of dissolved methane in CFAnMBR permeate (g/L), C_CH4=_methane percentage in headspace of vial, P = pressure (1 atm), K_CH4_ = Henry’s law constant at 25°C (1.410 × 10^−3^ mol/L-atm), MW_CH4_ = molecular weight of methane (16.043 g/mol), $V_{W}$ = volume of water (0.05 L), V_hs_ = volume of head-space in the vial (0.05 L), T_0_ = 273.15 K and Ta = 298.15 K.

The concentration of dissolved methane at thermodynamic equilibrium in the AFFB was computed with Henry’s law (Eq. 7):

$${CH}_{4,eq}=k_{H,CH4}\times P_{CH4}$$ (7)

Where ${CH}_{4,eq}$ = dissolved methane concentration at equilibrium (mg/L), $k_{H,CH4}$ : Henry’s law constant at 25°C for methane (1.410 × 10^-3^mol/L-atm), $P_{CH4}=$ the partial pressure of methane in the headspace of the CFAnMBR (atm). Methane was reported as measured at room temperature and not normalized to STP. Methane saturation index (the ratio of measured ${CH}_{4\left(aq\right)}$ to ${CH}_{4,eq}$ at thermodynamic equilibrium was assessed throughout the operational period. To estimate a COD mass balance of the CFAnMBR, daily measurements of headspace methane and effluent dissolved methane, along with composite samples of influent and effluent, were performed from Day 132—150.

### 2.4 Coagulation-flocculation process

Due to the larger pore size (5 µm) of the cloth filter membrane, it was hypothesized that adding a coagulation-flocculation process to the CFAnMBR system could improve TSS and COD removal efficiency. An inline coagulation-flocculation step was added downstream of the AFFB and prior to the cloth filter membrane. The inline coagulation-flocculation step consisted of a dosing pump (Masterflex L/S, Cole-Parmer, Vernon Hills, IL) and a static mixer (Koflo Corp., Cary, IL) in the AFFB effluent line. Ferric chloride (FeCl_3_) was injected immediately upstream of the static mixer. Coagulation-flocculation occurred in the cloth filter basin (99.6 L water-filled volume) upstream of the membrane, yielding a contact time of ∼24 min for the coagulation-flocculation process to occur.

The impact of adding either 50 mg/L or 100 mg/L of FeCl_3_ on effluent water quality and energy consumption was tested in two separate short-term trials lasting 28 h. The coagulant was dosed into the inlet pipe upstream of the static mixer which leads to the cloth-filter tank where coagulation and flocculation occurred. Permeate samples were collected every 2 h and analyzed for COD, TSS, and total P.

## 3 Results and discussion

### 3.1 TSS and COD removal efficiency

**FIGURE 2:**
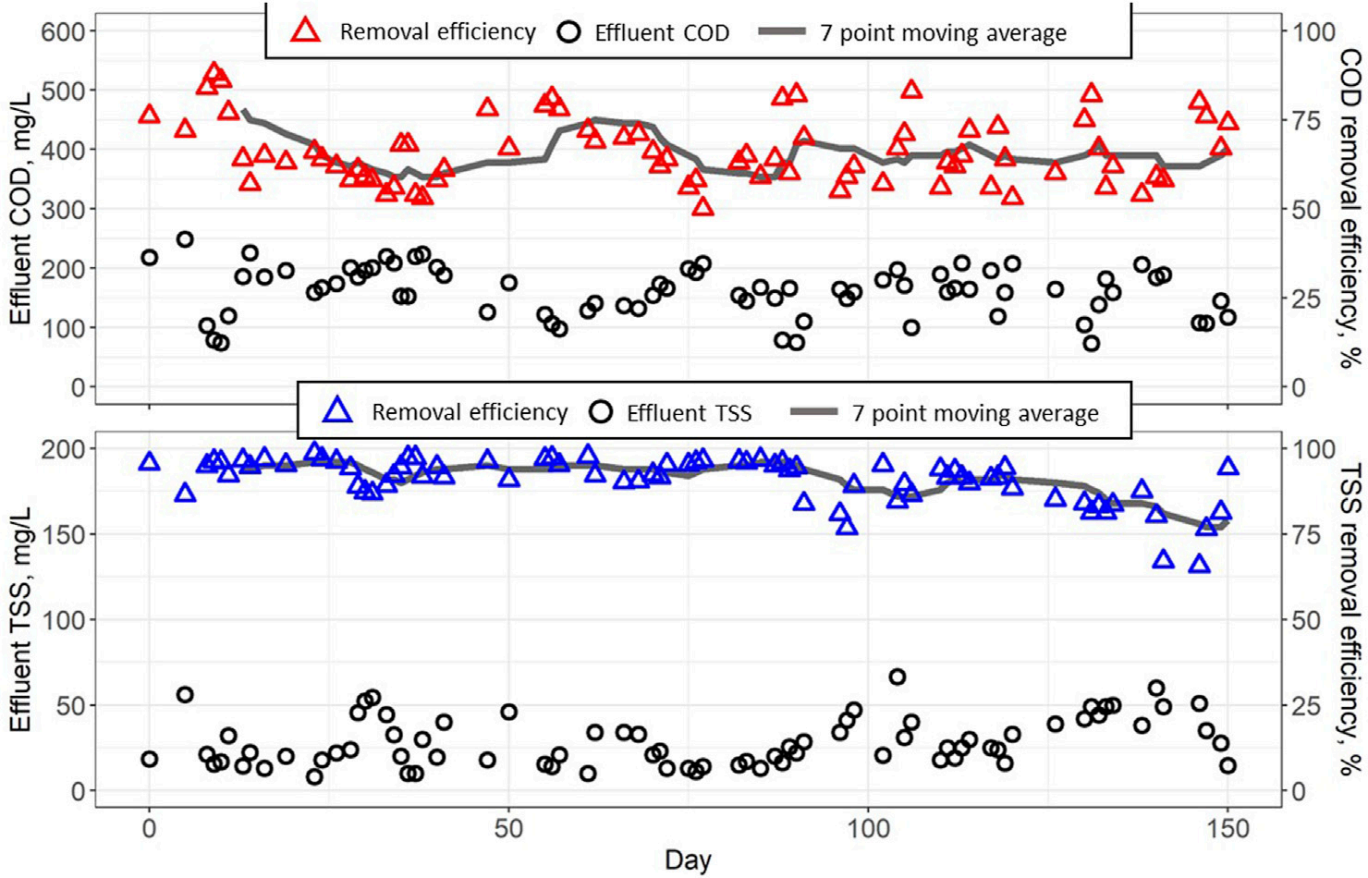
Effluent COD concentration and removal efficiency (top) and effluent TSS and removal efficiency (bottom) of the CFAnMBR from Day 0 (October 7) to Day 150 (March 6).

Start-up of the CFAnMBR on municipal wastewater occurred in October and over the first 150 days of operation, the average influent temperature was 13°C ± 4 °C (Table 2). Over this period, the average influent COD of the degritted municipal wastewater was 481 ± 90 mg COD/L, and the average organic loading rate (OLR) was 0.94 ± 0.20 kg COD/m^3^/d. Removal efficiency for COD was fairly stable over the entire period (Figure 2), with average COD removal efficiency of 66% ± 9%. The average effluent COD was 161 ± 41 mg/L, with soluble COD comprising 78% of remaining effluent COD. Removal efficiency remained relatively high as the biomass acclimated to the new source of influent organics and declining temperatures.

**TABLE 2:** Operating parameters and performance of the cloth filter anaerobic membrane bioreactor (CFAnMBR) treating municipal wastewater (Values include average ±standard deviation).

Time (days)	Influent temperature (°C)	Organic loading rate (kg COD/m^3^/day)	COD influent (mg/L)	COD effluent (mg/L)	sCOD effluent (mg/L)	COD removal efficiency (%)
0–37	16 ± 3	1.05 ± 0.20	541 ± 120	176 ± 46	129 ± 18	66 ± 10
38–61	14 ± 4	1.01 ± 0.12	515 ± 44	155 ± 49	118 ± 17	69 ± 10
62–85	12 ± 3	0.95 ± 0.06	443 ± 18	164 ± 24	137 ± 10	63 ± 6
86–150	11 ± 4	0.86 ± 0.22	449 ± 39	152 ± 39	121 ± 21	66 ± 9
0–150	13 ± 4	0.94 ± 0.20	481 ± 90	161 ± 41	126 ± 7	66 ± 9

By Day 38 after the transition to municipal wastewater, COD removal efficiency was consistently above 60% until Day 69, when the average outside air temperature decreased suddenly to −10°C and remained low from Day 70—78 during which time the influent water temperature dropped quickly from 16°C to 5°C. COD removal reduced to as low as 50% following this decrease in temperature, but then recovered and maintained an average COD removal of 66% from Day 86 to Day 150.

The majority of COD remaining in the effluent was soluble COD (sCOD), with an average effluent sCOD of 126 ± 7 mg/L (78% of effluent total COD). Effluent COD values exceeded the target of 60 mg COD/L to meet typical discharge limits for municipal wastewater treatment but was within the range of values reported in the literature for other AnMBRs operating at low temperatures. For instance, Gouveia et al. (2015a), working with a pilot submerged AnMBR treating municipal wastewater at 18°C ± 2°C, achieved a removal efficiency of 89.6% ± 2% with average effluent COD values around 120 mg/L at OLR between 1.5–2 kg COD/m^3^/day. Peña et al. (2019) reported COD effluent values between 100–130 mg/L when operating a pilot submerged AnMBR at 10°C with OLR 1–1.5 kg COD/m^3^/day. Gao et al. (2014), working with an anaerobic fluidized-bed membrane bioreactor treating domestic wastewater with an OLR of 1.44 kg COD/m^3^/day at 15°C, obtained a COD removal yield of 51.1% with an effluent COD of ∼170 mg/L. Watanabe et al. (2017) saw no significant difference in AnMBR permeate quality when the temperature was lowered from 25°C to 20°C, but significantly higher effluent COD (180 mg COD/L) when temperatures decreased from 20°C to 15°C.

Lim et al. (2019) observed COD removal >90% in an anaerobic digester operating at temperatures as low as 12.7°C, and Martinez-Sosa et al. (2011) achieved effluent COD concentrations below 80 mg/L and COD removal efficiency around 90% when treating synthetic wastewater at 15°C in an up-flow anaerobic sludge blanket (UASB) AnMBR. The high COD removal efficiency by Lim et al. and Martinez-Sosa et al. can be attributed to the use of an ultrafiltration membrane module with a relatively small pore size (0.038 µm) that retained all particulate COD and even some soluble COD. More comparable to the larger pore size of the current study, Zhang et al. (2010) operated a AnMBR with 61 µm pore-size filtration material and facilitated formation of a stable dynamic membrane layer (AnDMBR) for separation of particulate organics and microbes. The AnDMBR achieved COD removal of 57% ± 6% with an effluent COD concentration of 121 ± 34 mg/L and effluent TSS concentration below 15 mg/L at 10°C—15 °C.

These results highlight the effect of membrane pore size on COD removal efficiencies of AnMBRs, particularly under low temperature conditions. Decreased hydrolysis rate at low temperatures may contribute to higher colloidal COD concentration in the reactor, which ultra-filtration (UF) membranes are well-equipped to remove (Ozgun et al., 2015; Lim et al., 2019). In the present study, the cloth filter membrane had a larger pore size (nominal 5 µm) than most membranes used in previous AnMBR studies, which reduced COD removal efficiency. However, the COD removal was still significant and withing the range of other reported AnMBR results. The formation of a dynamic membrane layer on the cloth filter by the deposition of TSS, colloids, and biomass plays a key role in filtration by the CFAnMBR. Considering the cloth filter membrane did not fully retain all particulate nor soluble COD, and that the nominal pore size of the filter is at the upper range of cell size for methanogens (2—5 µm), it is likely that there would be some advantages for using a slightly lower pore size (1—2 µm). This would eliminate any potential loss of anaerobic microbes, while maintaining the permeability advantages for cloth filters versus most pervious AnMBR membranes.

Contrary to AnMBRs employing microfiltration (MF) and UF membranes in which complete TSS removal is observed (Martinez-Sosa et al., 2011; Shin et al., 2014; Ozgun et al., 2015; Lim et al., 2019), membranes with a larger pore size will likely have some particulate solids in the permeate. Even so, the CFAnMBR still had high TSS removal efficiency of 91% ± 7% with an average effluent TSS of 29 ± 14 mg/L. Concentrations as low as 8 mg TSS/L were measured in the effluent (Figure 2). Although MF and UF membrane bioreactors can achieve near complete TSS removal, this high removal efficiency is not required for anaerobic treatment considering that the most frequently used anaerobic system for mainstream wastewater treatment (i.e., UASB) has typical effluent TSS concentrations up to a few hundred mg/L (Tchobanoglous et al., 2014). Furthermore, the US EPA requires most municipal treatment plants to have a 30-day average effluent TSS <30 mg/L (USEPA, 2013). The pore size of MF and UF membranes is generally 10–100 times smaller than the anaerobic microbes that need to be retained in the process, which provides room for optimization when balancing microbe retention with the hydraulic performance of the membrane (e.g., flux). Most of the membranes used in previous AnMBRs were carried over from drinking water applications, and thus have not been completely optimized for the specific treatment goals and trade-offs in wastewater applications. The larger pore-size of the CFAnMBR offers the ability to reduce the cleaning energy and increase membrane flux compared to conventional MBRs, however, it also prevents this technology from realizing the disinfection benefits of conventional MBR. Thus, downstream disinfection may need to be applied in combination with the CFAnMBR.

While CFAnMBR effluent TSS and COD was above the municipal wastewater discharge limits, the technology could be used as an early step in wastewater treatment train for suspended solids and organic matter removal with minimal energy input and a significant biogas energy output. TSS and COD removal by the CFAnMBR exceeds typical removal efficiencies for primary clarifiers (60%—90% TSS removal, 30%—50% COD removal), indicating that effluent discharge limits would be possible with a very small aerobic treatment process after a CFAnMBR. Roughly 95% of facilities employing aerobic conventional activated sludge processes achieve an average effluent TSS below 20 mg/L (USEPA, 2013), which is generally achieved by sedimentation without use of a membrane or any other type of filter. A variety of post-treatment configurations to treat the effluent of anaerobic reactors have been reported in the literature, mainly investigating the treatment performance of different combinations of UASB and aerobic post-treatment systems, including trickling filter (TF), submerged aerated bio-filter (SABF) rotating biological contactor (RBC), constructed wetlands, sequencing batch reactor (SBR), chemically enhanced primary treatment (CEPT), zeolite column, and dissolved air flotation (DAF). According to (Khan et al., 2011), the complete removal of organic pollutants could be possible if the sewage could be treated via a sequential anaerobic, micro-aerobic, and fully aerobic biodegradation of the contaminants. By treating the majority of COD via anaerobic digestion, the CFAnMBR would significantly reduce aeration energy inputs during typical secondary treatment. Although effluent ammonia was not measured over the study period, subsequent monitoring of the CFAnMBR showed that effluent ammonia was not significantly different from influent ammonia, indicating a downstream ammonia removal process would be required.

### 3.2 Membrane cleaning energy demand and hydraulic flux

**FIGURE 3:**
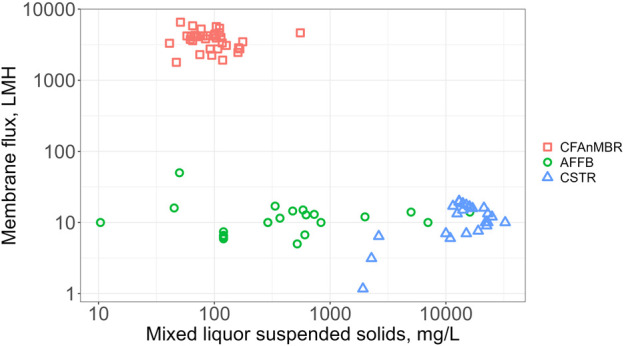
Flux rates (LMH, L/m^2^/h) versus mixed liquor suspended solids for AnMBRs with anaerobic fixed-film bioreactors (AFFBs) and completely stirred tank reactors (CSTRs) compared to the cloth filter anaerobic membrane bioreactor (CFAnMBR) from this study.

The CFAnMBR improved membrane flux and membrane cleaning energy to counteract fouling, which are two important parameters that previously limited the economic feasibility of AnMBR for municipal wastewater treatment. Measured values of membrane flux for the CFAnMBR ranged from 1,385—6542 LMH, with an average and standard deviation of 3,649 ± 1246 LMH (Figure 3). Membrane flux of the CFAnMBR was greater than reported values for previous AnMBR configurations (∼10—20 LMH) by more than two orders of magnitude (Giménez et al., 2011; Martinez-Sosa et al., 2011; Giménez et al., 2014; Shin et al., 2014; Awasthi et al., 2016; Li and Yu, 2016; Diez et al., 2021). This represents a significant reduction in required size of the membrane system for mainstream anaerobic digestion. These high fluxes were made possible by two main factors: 1) the larger membrane pore size (nominal 5 µm) and 2) the use of plastic biofilm support media prior to the cloth filter that reduced solids loading impinging on the filter.

Mixed liquor suspended solids (MLSS) in a MBR bioreactor has been reported as a critical factor affecting fouling in typical MF/UF MBRs, and higher MLSS generally decreases the flux rate and/or increases the cleaning energy demand for to maintain the flux (Dong et al., 2016). The earliest and most common AnMBR systems consisted of a completely stirred tank reactor (CSTR) with a membrane at the outlet to retain biosolids (Kim et al., 2011). In this configuration, the concentration of solids impinging on the membrane is the same as the CSTR mixed liquor, which is usually very high (10–30 g/L, Liao et al., 2006). The current study used an anaerobic fixed-film design, and other recent bench-scale studies have used various AFFB configurations (e.g., UASB or fluidized bed) with a membrane system at the AFFB outlet (Kim et al., 2011; Shin et al., 2014; Seib et al., 2016a; Seib et al., 2016b). These studies have shown that reduced solids impinging on the membrane allows for higher membrane flux rates than CSTR style MBRs, but these flux rates are still much lower than those demonstrated in the current study on CFAnMBRs. For instance, Aslam et al. (2014) achieved flux rates of 50 LMH when working with an anaerobic fluidized bed membrane reactor (AFMBR).

While the CFAnMBR showed significantly higher membrane flux relative to CSTR AnMBRs using much higher MLSS, similar increases were also seen relative to previously reported AFFB AnMBR with comparable MLSS. This was attributed to the larger pore size of the cloth filter membrane, consistent with findings that the membrane pore size significantly affects membrane flux (Liao et al., 2006). A clear relationship between MLSS and membrane flux for the CFAnMBR was not observed, and flux rates remained high even during extreme solids loading rates. For instance, during CFAnMBR start-up, MLSS impinging on the cloth filter reached >550 mg/L while membrane flux remained high (4640 LMH). This flux was still orders of magnitude greater than flux rates reported for previous AFFB AnMBR (generally 5–50 LMH).

**FIGURE 4:**
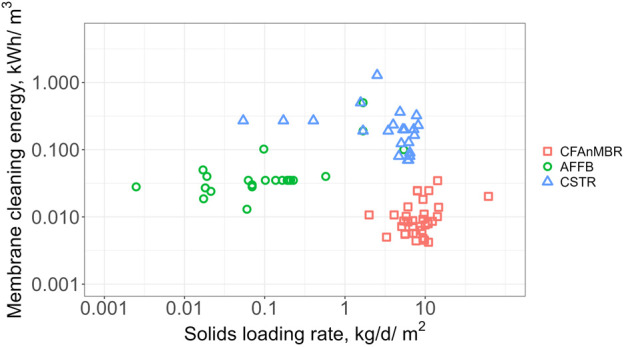
Membrane **c**leaning energy demand versus solids loading rate for AnMBRs with anaerobic fixed-film bioreactors (AFFBs) and completely stirred tank reactors (CSTRs) compared to the cloth filter anaerobic membrane bioreactor (CFAnMBR) from this study.

Membrane cleaning energy was also reduced relative to prior AnMBR configurations as shown in Figure 4. The cloth filter membrane resulted in membrane cleaning energy requirements ranging from 0.0042—0.035 kWh/m^3^, with a median value of 0.0085 kWh/m^3^. Relative to membrane cleaning energy reported in prior AnMBR studies, the CFAnMBR median membrane cleaning energy was 96% lower than the CSTR median (0.20 kWh/m^3^) and 76% lower than the AFFB median (0.035 kWh/m^3^). Similar low energy AnMBR configurations resulted in cleaning energy demands between 0.04 kWh/m^3^ for a pilot UASB-AnMBR (Gouveia et al., 2015a) and 0.10 kWh/m^3^ for a pilot two-stage AnMBR using hollow fiber membranes (Shin et al., 2014). These configurations achieved low solids concentrations in the membrane tank using recirculation of liquid between the bioreactor and membrane tank and periodic withdrawal of solids from the membrane tank. The CFAnMBR membrane cleaning energy is substantially lower than state-of-the-art aeration-based organics removal for municipal wastewater (e.g., CAS) with typical energy demand of 0.3–0.6 kWh/m^3^ (Tchobanoglous et al., 2014).

Since the CFAnMBR operates at much higher flux rates than other AnMBR configurations, a lower cleaning energy demand per volume of treated water should be expected. To compare the CFAnMBR energy demand to reported values for prior AnMBRs, cleaning energy demand was plotted as a function of solids loading rate, or the TSS concentration multiplied by the permeate flux (Figure 4). As noted above, the CFAnMBR had 96% lower median cleaning energy demand relative to previous CSTR AnMBR that operate at similar solids loading rates. The CFAnMBR also reduced cleaning energy demand relative to AFFB AnMBR, which is made possible by the large pore size of the cloth membrane. The CFAnMBR utilizes the benefits of the AFFB design (i.e., reducing impinging solids) while also allowing a high flux to provide high solids loading capacity on the filter.

The energy demand for fouling control in AnMBR systems depends on several factors: membrane type, pore size, bioreactor configuration, fouling control method, flux rate, and the solids loading impinging on the membrane surface. High solids loading increases membrane fouling and the amount of energy used for fouling control—conditions that previously limited the use of AnMBRs for full-scale municipal wastewater treatment. Previous studies showed that reducing the solids loading from up to 30 g/L in CSTR systems to less than 5 g/L in fixed-film AnMBRs decreased the energy consumption for fouling control from between 0.07—1.35 kWh/m^3^ to between 0.02—0.50 kWh/m^3^ (Kim et al., 2011; Aslam et al., 2014; Ren et al., 2014; Shin et al., 2014; Gouveia et al., 2015a; Gouveia et al., 2015b; Pretel et al., 2015; Seib et al., 2016a; Seib et al., 2016b; Pretel et al., 2016; Shin and Bae, 2018; Liu et al., 2020). The AnMBR configurations mostly rely on submerged membranes that use backwashing and gas or particle sparging as the primary fouling control method. The CFAnMBR relied on a vacuum shoe for backwashing that manages fouling of the more porous cloth membrane, which is the method used in commercial installations at municipal wastewater facilities for tertiary treatment.

### 3.3 Biogas production

**FIGURE 5:**
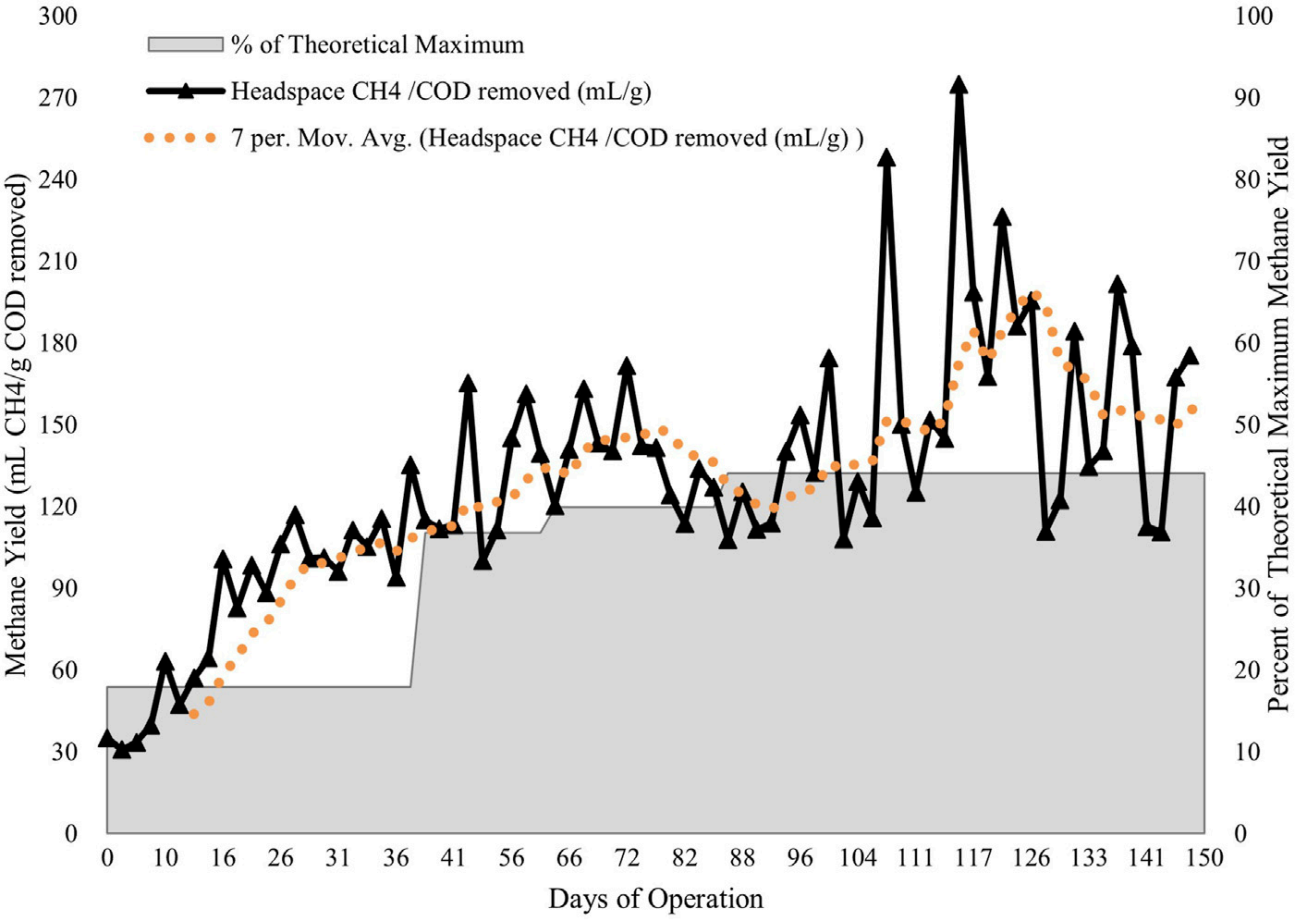
Headspace methane production in the fixed-film bioreactor tank of the CFAnMBR over the study period along with the percent of the theoretical maximum of COD conversion to methane (350 mL CH_4_/g COD_removed_).

Robust biogas production was observed by the end of the 150 days study period (Figure 5). Headspace methane yield during the first 37 days (81 ± 31 mL CH_4_/g COD_removed_) was not considered to be indicative of longer-term system performance as it represented the biomass acclimation following the transition from intermittent, synthetic wastewater to continuous, municipal wastewater with an influent temperature below 20 °C. Biogas production steadily improved and eventually averaged 154 ± 41 mL CH_4_/g COD_removed_ for Days 86—150, which is in the range of values reported in the literature for anaerobic reactors under psychrophilic conditions (0°C–20°C).

Methane yield remained robust even as temperatures fell from 16°C ± 3 °C during Days 0—37 to 11°C ± 4 °C during Days 86—150. Methane yield was still increasing toward the end of the 150 days period even as temperatures further decreased through the winter, suggesting that the microbial community was still acclimating in the bioreactor. The microbial community had been acclimated at ambient temperature over 12 months before deployment in the CFAnMBR, however the transition to municipal wastewater introduced new environmental variables that required additional acclimation. Stable biogas production even at low HRT (∼12.8 h) was made possible by retention of organics by the membrane and the subsequent return to the fixed film bioreactor during cloth filter backwashing, resulting in sufficient SRT for anaerobic digestion. The sustained biogas production over the study period suggests that the cloth filter membrane and biofilm support media were successful in preventing the rate of biomass loss from exceeding microbial growth rates. Hydrolysis, not methanogenesis, has been demonstrated to be the typical limiting step in anaerobic processes at low temperatures because it is more temperature-sensitive than methanogenesis (Ribera-Pi et al., 2020).

Average methane content of the biogas was 59% ± 9% over the study period, comparable to biogas methane content reported for AnMBR. Towards the end of the study period (Day 86—150), the average values of biogas composition were 62% ± 10% CH_4_, 37% ± 8% CO_2_, 38 ± 12 ppm H_2_S. Average methane yield increased to 154 mL CH_4_/gCOD_removed_ by Day 150 (11°C ± 4 °C). This study reports methane yield as mL CH/g COD_removed_, regardless of the mechanism, since COD removal can occur through mechanisms other than methanogenesis (e.g., sulfate reduction). Biogas production was lower than other AnMBR studies operating at temperatures of 25 °C (220—270 mL CH_4_/g COD_removed_; Kong et al., 2021; Sanchez et al., 2022), but comparable to Gao et al. (2014) operating an AnMBR at 15 °C with methane yield of 140 mL CH_4_/g COD_removed_. Reeduced temperatures below 20 °C can significantly affect the potential amount collectable headspace biogas because reduced microbial metabolism rates as well as increases in methane solubility.

A COD mass balance for the CFAnMBR was conducted for Days 132—150. Headspace methane accounted for 22% of COD entering the system. Based on measurements of dissolved methane in the effluent, it accounted for 19% of the COD entering the system. Previous studies have reported that at temperatures below 15°C, 40%—60% of methane produced in AnMBR can leave as dissolved methane at supersaturated conditions in the effluent (Shin et al., 2014). If dissolved methane were considered in the biogas balance, a methane yield of 310 mL CH_4_/g COD_removed_ would be obtained, which is still lower than the theoretical value. Based on H_2_S content of the biogas, approximately 6% of the influent COD was used for sulfate reduction, similar to findings by Shin et al. (2014). The particulate and soluble organics that were not completely degraded but retained by the AnMBR system accounted for 42% of the influent COD, and 11% of the influent COD was losses or otherwise not accounted for in the mass balances. The amount of undegraded organics is expected to be higher during the winter season, and assuming sufficient room for storage in the bioreactor, those organics could be retained until warmer temperatures return and biodegradation rates increase.

Reducing dissolved methane in AnMBR effluent and subsequent direct greenhouse gas emissions is necessary for industry adoption. Conventional sidestream anaerobic digestion operates at mesophilic temperatures, while mainstream anaerobic digestion requires operation at ambient temperatures to avoid excessive process heat inputs. Dissolved methane emissions increase as temperatures decrease (Crone et al., 2016). The CFAnMBR does not address the issue of dissolved methane in AnMBR effluent and this topic was not included in the experimental work for this study. From other literature on this subject, the most common strategies for dissolved methane removal are aeration, gas stripping, biological methane oxidation, and degassing membrane (Hatamoto et al., 2010; Velasco et al., 2018). Methane can be biologically oxidized by methanotrophs, with reported removal efficiencies of up to 95% (Hatamoto et al., 2010). Degassing membranes provide the good potential for dissolved methane recovery due to their ease of operation and high mass transfer area (Rongwong et al., 2017), while agitation provides the lowest methane recovery among the technologies listed above. Sparging and degassing membrane produce the best methane recovery with medium to high capital and operating costs (Velasco et al., 2018), and vacuum degassing methods have been shown to produce more energy in recovered methane than expended for degassing (Lee et al., 2020).

### 3.4 Coagulation-flocculation for improving CFAnMBR effluent quality

**FIGURE 6:**
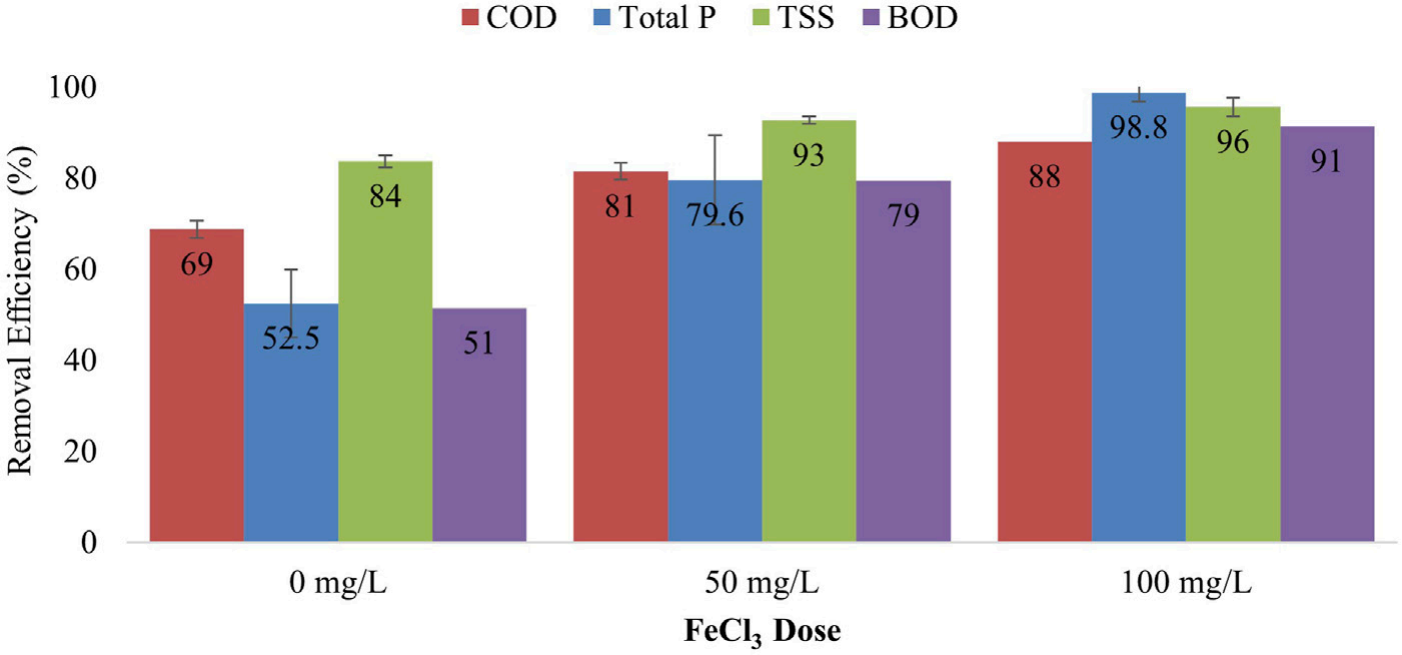
Effect of in-line coagulant dosing using FeCl_3_ (50 and 100 mg/L) on effluent quality in terms of COD, TSS, Total P, and BOD removal in the pilot CFAnMBR. (Error bars represent standard deviation).

Short-term experiments using in-line, coagulation tests with FeCl_3_ demonstrated the ability to increase COD and TSS removal efficiency with the CFAnMBR. The addition of 50 mg/L of FeCl_3_ increased average removal of COD, Total P, TSS, and BOD by 12, 27, 9, and 28 percentage points, respectively (Figure 6). The effect of the coagulant addition was further increased at higher dosage rates. The addition of 100 mg/L of FeCl_3_ increased COD, Total P, TSS, and BOD removal by 19, 46, 12, and 40 percentage points, respectively, in comparison to operations without coagulant addition.

Similar results were reported by Jaya Prakash et al. (2007) employing a coagulation-flocculation process to treat UASB effluent, reducing BOD and TSS concentrations from 38 to 55 and 65–110 mg/L, respectively, to less than 20 mg/L and 50 mg/L using 110 mg/L FeCl_3_. Aiyuk et al. (2004) proposed an integrated coagulation-flocculation–UASB-zeolite column concept for the low-cost treatment of domestic wastewater. In this integrated treatment system, domestic wastewater was initially subjected to chemically enhanced primary treatment (CEPT) using 50 mg/L FeCl_3_ as a coagulant and a polymer to remove suspended material and phosphorus, followed by UASB treatment to remove soluble organics, showing removal efficiencies of 73% COD, 85% TSS, and 80% PO_4_^3−^. However, the coagulation-flocculation step placed before the UASB produced thick sludge containing 8.4% solids that still needed to be treated and disposed of. By placing the coagulation-flocculation process between the anaerobic reactor and the cloth-filter tank in the current study, the need for additional tankage for digestion of sludge was eliminated as the sludge produced was already biologically stabilized in the CFAnMBR.

Another advantage of the coagulation step is that the addition of FeCl_3_ has been shown to reduce irreversible fouling on AnMBRs by increasing particle sizes and reducing the colloidal/soluble substances in the mixed liquor (Dong et al., 2015). Additionally, since the chemicals are recirculated back to the reactor during backwash and solids waste events, the optimum FeCl_3_ dosage may be reduced over time as some FeCl_3_ is accumulated in the system. This hypothesis, however, needs to be confirmed with further study. Optimization of this process could include the use of other coagulation/flocculation agents to improve efficiency, such as cationic starch polymers that would be biodegradable when the filter backwash is recycled back to the fixed-film anaerobic bioreactor.

The results presented in this study represent short-term effects of coagulant dosing to demonstrate ability to improve CFAnMBR COD removal efficiency. Subsequent work would determine the effect on COD removal efficiency under long-term coagulant addition at optimized dosing rates. Trade-offs related to increased sludge production and coagulant cost under optimized dosing rates need to be further investigated. Nonetheless, it is worth noting that even with the proposed level of FeCl_3_ addition, the overall sludge yields for the AnMBR process are less than those associated with conventional aerobic wastewater treatment. According to Tchobanoglous et al. (2014), the conventional activated sludge process typically produces 80 g of dry solids per m^3^ of wastewater treated. In addition, about 150 g/m^3^ of primary sludge is produced in the primary sedimentation tanks used in most activated sludge configurations, for a total of ∼230 g/m^3^ dry sludge produced in CAS. Conversely, total amount of sludge produced by the CFAnMBR is 93 g/m^3^ is removed without coagulation. When 50 mg/L or 100 mg/L of FeCl_3_ are dosed, the sludge production increases to 157 g/m^3^ and 201 g/m^3^, respectively.

### 3.5 Facilitating AnMBR adoption for municipal wastewater

The CFAnMBR reduced median energy demand for membrane fouling control by 76%—96% relative to prior AnMBR configurations. This represents a significant reduction in operating expenses related to energy consumption during the membrane cleaning step, which has previously been recognized as a significant cost barrier to adoption of AnMBR technology for the application of municipal wastewater (Aslam et al., 2022). Reductions in capital expenses are also significant. The per unit area cost of the cloth filter membrane is lower than other AnMBR membranes. Estimated cost of the cloth filter membrane material is $32/m^2^ (personal communication with manufacturer, 2020). Lin et al. (2011) reported membrane cost of $42/m^2^, converted to 2020 US dollars, and Pretel et al. (2016) reported cost for a UF hollow-fiber membrane of $58/m^2^. The majority of the membrane cost savings, however, come from the increase in membrane flux by > 2 orders of magnitude, resulting in a proportional decrease in required membrane area. Lin et al. (2011) found that membrane costs using conventional membranes accounted for 46%—72% of total capital costs of MBR systems.

While full-scale AnMBR of various configurations have been successfully deployed in commercial operations for industrial wastes (e.g., food and beverage, animal waste), use of AnMBRs in these applications is feasible due to the higher-strength wastewater (COD >1,000 mg/L) and lower hydraulic loads. Conversely, municipal wastewater treatment requires higher hydraulic capacity for relatively low strength wastewater (COD 300—500 mg/L). Adoption of AnMBR technology in the municipal wastewater industry has thus far been limited for this reason. The CFAnMBR resolves a major limitation to industry adoption by increasing membrane flux and increasing the solids loading rate capabilities of the system.

Further, the cloth filter technology utilizes a commercially available membrane with existing adoption and technical experience in the municipal wastewater industry. There are several manufacturers and equipment providers of cloth filter technology in the US. The equipment and operational modifications necessary for using the cloth filter technology for treating anaerobic digester effluent are comparatively minor barriers to adoption, relative to the modifications and scale-up required for current AnMBR membranes which have yet to resolve the high capital and operational expenses related membrane cost and fouling control.

## 4 Conclusion

The novel cloth filter anaerobic membrane bioreactor (CFAnMBR) resolves two major limitations of AnMBR use for municipal wastewater treatment. The CFAnMBR increased membrane flux by greater than two orders of magnitude (3,649 ± 1,246 LMH) and reduced cleaning energy demand by 76%—96% (0.0085 kWh/m^3^) relative to previously reported AnMBR configurations. The tradeoff of higher membrane flux and lower cleaning energy due to larger membrane pore size was lower COD and TSS removal (66% and 91%, respectively). The removal efficiency for these parameters was improved by the use of ferric chloride to promote in-line coagulation-flocculation, which increased COD and TSS removal to 81%—88% and 93%—96%. Subsequent study of the CFAnMBR will investigate long-term performance of the system in terms of COD removal and biogas production, with the goal of improving COD removal efficiency, and to bring AnMBR effluent levels closer to the discharge limit for municipal wastewater. Preliminary technoeconomic comparison of the CFAnMBR shows that it will significantly reduce both capital and operating costs in comparison to previous AnMBR configurations. Thus, the CFAnMBR provides a pathway for expediting AnMBR adoption in the WRRF industry by re-purposing a commercially available cloth filter technology currently used for municipal wastewater treatment, although some equipment and operational modifications are likely necessary to accommodate AnMBR applications.

## Data Availability

The raw data supporting the conclusion of this article will be made available by the authors, without undue reservation.
